# PReS-FINAL-2318: The structural and functional changes of biomembrains of FMF and HSP with children

**DOI:** 10.1186/1546-0096-11-S2-P308

**Published:** 2013-12-05

**Authors:** H Sargsyan, P Ghazaryan

**Affiliations:** 1 Yerevan State Medical University, Yerevan, Armenia; 2 Haematological Centre, Yerevan, Armenia

## Introduction

According to some clinical feature of similarities (fever, abdominal pain, arthritis, skin manifestation, gastro-intestinal disorders and kidney diseases) Familial Mediterranean Fever (FMF) and Henoch-Schonlein Purpura (HSP) we attempt to identify membranes aspects of pathogeneses these diseases.

## Objectives

The aim of our study is to identify some changes of individual phospholipids (PL) in erythrocyte membranes and comparative analysis of FMF and HSP patients.

## Methods

The examinations have carried out in 42 non complicated 7-15 ages of FMF patients and 24 healthy volunteers. Clinical studies have carried out in the National FMF Children Center, Center "Arabkir". Biochemical changes have studied in Hematological Center. 42 patients in erythrocyte membranes by the separate individual PL are studied: phosphatidylcholines (PCH), phosphatidylethanolamines (PE), phosphatidylinosites (PI), sphingomyelins (SPM), phosphatidic acids (PA), phosphatidylserine (PS) and citotoxic-LysoPCH (LPCH) qualitative and quantitative changes. Previously it has been investigated the changes of indicators during the HSP diseases (L. Simonyan et al., 2011). The fractions of separate PL in erythrocyte membranes are carried out by thin-layer chromatography methods and decay their enzymes (phospholipase A) activity was determined by the spektrofotometrik method.

## Results

Comparative studies are identified on the basis of membrane structural metabolism disorders, for the prevention and regulation some changes of FMF and HSP. Based on the results obtained by our research is an attempt to separate the membrane lipids metabolism disorder specific agencies which are responsible for affection of membrane's.

## Conclusion

According to our investigation we can conclude, that level of phopholipase A and citotoxic-LPCH are increase in mentioned diseases.

## Disclosure of interest

None declared.

